# Supplementary material for: Resolving the full spectrum of human genome variation using Linked-Reads
Source: Genome Res. 2019 Apr;29(4):635–45. doi: 10.1101/gr.234443.118 (PMC6442396; doi:10.1101/gr.234443.118)
Supplement: Supplemental Material [file supp_gr.234443.118_Supplemental_longranger-2.2.zip › longranger-2.2.tar.bz2!/lr2.2-public/lib/go/src/code.google.com/vitess/docs/overview/scaling-mysql.html]

Vitess / Understanding Vitess 


Toggle navigation

- Overview
- Guides
- Reference
- Blog
- GitHub

- #### Overview

  - What is Vitess
  - Scaling MySQL with Vitess
  - Key Concepts
- #### Getting Started

  - Run Vitess on Kubernetes
    - Custom Docker Build
  - Run Vitess Locally
- #### User Guide

  - Introduction- Client Libraries- Backing Up Data- Reparenting
        - Schema Management
        - VSchema Guide
        - Schema Swap (Tutorial)
        - Sharding
          - Horizontal Sharding (Tutorial, automated)
          - Horizontal Sharding (Tutorial, manual)
          - Sharding in Kubernetes (Tutorial, automated)
          - Sharding in Kubernetes (Tutorial, manual)
        - Vitess Sequences
        - MySQL Server Protocol
        - Vitess and Replication
        - Update Stream
        - Row Based Replication
        - Topology Service
        - Transport Security Model
        - Launching
          - Scalability Philosophy
          - Production Planning
          - Server Configuration
          - 2PC Guide
          - Troubleshooting
        - Upgrading
- #### Reference Guides

  - Vitess API- vtctl Commands
- #### Other Resources

  - Presentations- Blog- Roadmap
- #### Contributing

  - Contributing to Vitess- GitHub Workflow- Code Reviews
- GitHub

# Understanding Vitess

- #### Overview

  - What is Vitess
  - Scaling MySQL with Vitess
  - Key Concepts
- #### Getting Started

  - Run Vitess on Kubernetes
    - Custom Docker Build
  - Run Vitess Locally
- #### User Guide

  - Introduction- Client Libraries- Backing Up Data- Reparenting
        - Schema Management
        - VSchema Guide
        - Schema Swap (Tutorial)
        - Sharding
          - Horizontal Sharding (Tutorial, automated)
          - Horizontal Sharding (Tutorial, manual)
          - Sharding in Kubernetes (Tutorial, automated)
          - Sharding in Kubernetes (Tutorial, manual)
        - Vitess Sequences
        - MySQL Server Protocol
        - Vitess and Replication
        - Update Stream
        - Row Based Replication
        - Topology Service
        - Transport Security Model
        - Launching
          - Scalability Philosophy
          - Production Planning
          - Server Configuration
          - 2PC Guide
          - Troubleshooting
        - Upgrading
- #### Reference Guides

  - Vitess API- vtctl Commands
- #### Other Resources

  - Presentations- Blog- Roadmap
- #### Contributing

  - Contributing to Vitess- GitHub Workflow- Code Reviews

Traditionally, it's been difficult to scale a MySQL-based database to an arbitrary size. Since MySQL lacks the out-of-the-box multi-instance support required to really scale an application, the process can be complex and obscure.

As the application grows, scripts emerge to back up data, migrate a master database, or run some offline data processing. Complexity creeps into the application layer, which increasingly needs to be aware of database details. And before we know it, any change needs a big engineering effort so we can keep scaling.

Vitess grew out of YouTube's attempt to break this cycle, and YouTube decided to open source Vitess after realizing that this is a very common problem. Vitess simplifies every aspect of managing a MySQL cluster, allowing easy scaling to any size without complicating your application layer. It ensures your database can keep up when your application takes off, leaving you with a database that is flexible, secure, and easy to mine.

This document talks about the process of moving from a single small database to a limitless database cluster. It explains how steps in that process influenced Vitess' design, linking to relevant parts of the Vitess documentation along the way. It concludes with tips for designing a new, highly scalable application and database schema.

## Getting started

Vitess sits between your application and your MySQL database. It looks at incoming queries and routes them properly. So, instead of sending a query directly from your application to your database, you send it through Vitess, which understands your database topology and constantly monitors the health of individual database instances.

While Vitess is designed to manage large, multi-instance databases, it offers features that simplify database setup and management at all stages of your product's lifecycle.

Starting out, our first step is getting a simple, reliable, durable database cluster in place with a master instance and a couple of replicas. In Vitess terminology, that's a single-shard, single-keyspace database. Once that building block is in place, we can focus on replicating it to scale up.

### Planning for scale

We recommend a number of best practices to facilitate scaling your database as your product evolves. You might not experience the benefits of these actions immediately, but adopting these practices from day one will make it much easier for your database and product to grow:

- Always keep your database schema under source control and provide unit test coverage of that schema. Also check schema changes into source control and run unit tests against the newly modified schema.
- Think about appropriate sharding keys for your data and structure that data accordingly. Usually, sharding keys are obvious -- e.g. a user ID. However, having your key(s) in place ahead of time is much easier than needing to retrofit your data before you can actually shard it.
- Group tables that share the same sharding key. Similarly, split tables that don’t share the same sharding key into different keyspaces.
- Avoid cross-shard queries (scatter queries). Instead, use MapReduce for offline queries and build Spark data processing pipelines for online queries. Usually, it is faster to extract raw data and then post-process it in the MapReduce framework.
- Plan to have data in multiple data centers and regions. It is easier to migrate to multiple data centers if you've planned for incoming queries to be routed to a region or application server pool that, in turn, connects to the right database pool.

## Step 1: Setting up a database cluster

At the outset, plan to create a database cluster that has a master instance and a couple of read-only replicas (or slaves). The replicas would be able to take over if the master became unavailable, and they might also handle read-only traffic. You'd also want to schedule regular data backups.

It's worth noting that master management is a complex and critical challenge for data reliability. At any given time, a shard has only one master instance, and all replica instances replicate from it. Your application -- either a component in your application layer or Vitess, if you are using it -- needs to be able to easily identify the master instance for write operations, recognizing that the master might change from time to time. Similarly, your application, with or without Vitess, should be able to seamlessly adapt to new replicas coming online or old ones being unavailable.

### Keep routing logic out of your application

A core principle underlying Vitess' design is that your database and data management practices should always be ready to support your application's growth. So, you might not yet have an immediate need to store data in multiple data centers, shard your database, or even do regular backups. But when those needs arise, you want to be sure that you'll have an easy path to achieve them. Note that you can run Vitess in a Kubernetes cluster or on local hardware.

With that in mind, you want to have a plan that allows your database to grow without complicating your application code. For example, if you reshard your database, your application code shouldn't need to change to identify the target shards for a particular query.

Vitess has several components that keep this complexity out of your application:

- Each MySQL instance is paired with a **vttablet** process, which provides features like connection pooling, query rewriting, and query de-duping.
- Your application sends queries to **vtgate**, a light proxy that routes traffic to the correct vttablet(s) and then returns consolidated results to the application.
- The **Topology Service** -- Vitess supports Zookeeper and etcd -- maintains configuration data for the database system. Vitess relies on the service to know where to route queries based on both the sharding scheme and the availability of individual MySQL instances.
- The **vtctl** and **vtctld** tools offer command-line and web interfaces to the system.

Setting up these components directly -- for example, writing your own topology service or your own implementation of vtgate -- would require a lot of scripting specific to a given configuration. It would also yield a system that would be difficult and costly to support. In addition, while any one of the components on its own is useful in limiting complexity, you need all of them to keep your application as simple as possible while also optimizing performance.

**Optional functionality to implement**

- *Recommended*. Vitess has basic support for identifying or changing a master, but it doesn't aim to fully address this feature. As such, we recommend using another program, like Orchestrator, to monitor the health of your servers and to change your master database when necessary. (In a sharded database, each shard has a master.)
- *Recommended*. You should have a way to monitor your database topology and set up alerts as needed. Vitess components facilitate this monitoring by exporting a lot of runtime variables, like QPS over the last few minutes, error rates, and query latency. The variables are exported in JSON format, and Vitess also supports an InfluxDB plug-in.
- *Optional*. Using the Kubernetes scripts as a base, you could run Vitess components with other configuration management systems (like Puppet) or frameworks (like Mesos or AWS images).

**Related Vitess documentation:**

- Running Vitess on Kubernetes
- Running Vitess on a local server
- Backing up data
- Reparenting - basic assignment of master instance in Vitess

## Step 2: Connect your application to your database

Obviously, your application needs to be able to call your database. So, we'll jump straight to explaining how you'd modify your application to connect to your database through vtgate.

### Using the Vitess connector

The main protocol for connecting to Vitess is gRPC. The connection lets the application see the database and send queries to it. The queries are virtually identical to the ones the application would send directly to MySQL.

Vitess supports connections for several languages:

- **Go**: We provide a sql/database driver.
- **Java**: We provide a library that wraps the gRPC code and a JDBC driver.
- **PHP**: We provide a library that wraps the gRPC code and a PDO driver.
- **Python**: We provide a library that wraps the gRPC code and a PEP-compliant driver.

#### Unit testing database interactions

The vttest library and executables provide a unit testing environment that lets you start a fake cluster that acts as an exact replica of your production environment for testing purposes. In the fake cluster, a single DB instance hosts all of your shards.

### Migrating production data to Vitess

The easiest way to migrate data to your Vitess database is to take a backup of your existing data, restore it on the Vitess cluster, and go from there. However, that requires some downtime.

Another, more complicated approach, is a live migration, which requires your application to support both direct MySQL access and Vitess access. In that approach, you'd enable MySQL replication from your source database to the Vitess master database. This would allow you to migrate quickly and with almost no downtime.

Note that this path is highly dependent on the source setup. Thus, while Vitess provides helper tools, it does not offer a generic way to support this type of migration.

**Related Vitess documentation:**

- Vitess API Reference
- Schema Management
- Transport Security Model

## Step 3: Vertical sharding (scaling to multiple keyspaces)

Typically, the first step in scaling up is vertical sharding, in which you identify groups of tables that belong together and move them to separate keyspaces. A keyspace is a distributed database, and, usually, the databases are unsharded at this point. That said, it's possible that you'll need to horizontally shard your data (step 4) before scaling to multiple keyspaces.

The benefit of splitting tables into multiple keyspaces is to parallelize access to the data (increased performance), and to prepare each smaller keyspace for horizontal sharding. And, in separating data into multiple keyspaces, you should aim to reach a point where:

- All data inside a keyspace scales the same way. For example, in an e-commerce application, user data and product data don’t scale the same way. But user preferences and shopping cart data usually scale with the number of users.
- You can choose a sharding key inside a keyspace and associate each row of every table with a value of the sharding key. Step 4 talks more about choosing a sharding key.
- Joins are primarily within keyspaces. (Joins between keyspaces are costly.)
- Transactions involving data in multiple keyspaces, which are also expensive, are uncommon.

### Scaling keyspaces with Vitess

Several vtctl functions -- vtctl is Vitess' command-line tool for managing your database topology -- support features for vertically splitting a keyspace. In this process, a set of tables can be moved from an existing keyspace to a new keyspace with no read downtime and write downtime of just a few seconds.

**Related Vitess documentation:**

- vtctl Reference guide

## Step 4: Horizontal sharding (partitioning your data)

The next step in scaling your data is horizontal sharding, the process of partitioning your data to improve scalability and performance. A shard is a horizontal partition of the data within a keyspace. Each shard has a master instance and replica instances, but data does not overlap between shards.

In general, database sharding is most effective when the assigned keyspace IDs are evenly distributed among shards. Keyspace IDs identify the primary entity of a keyspace. For example, a keyspace ID might identify a user, a product, or a purchase.

Since vanilla MySQL lacks native sharding support, you'd typically need to write sharding code and embed sharding logic in your application to shard your data.

### Sharding options in Vitess

A keyspace in Vitess can have three sharding schemes:

- **Not sharded** (or **unsharded**). The keyspace has one shard, which contains all of the data.
- **Custom**: The keyspace has multiple shards, each of which can be targeted by a different connection pool. The application needs to target statements to the right shards.
- **Range-based**: The application provides a sharding key for each record, and each shard contains a range of sharding keys. Vitess uses the sharding key values to route queries to the right shards and also supports advanced features like dynamic resharding.

A prerequisite for sharding a keyspace in Vitess is that all of the tables in the keyspace contain a keyspace ID, which is a hashed version of the sharding key. Having all of the tables in a keyspace share a keyspace ID was one of the goals mentioned in section 3, but it's a requirement once you're ready to shard your data.

Vitess offers robust resharding support, which involves updating the sharding scheme for a keyspace and dynamically reorganizing data to match the new scheme. During resharding, Vitess copies, verifies, and keeps data up-to-date on new shards while existing shards continue serving live read and write traffic. When you're ready to switch over, the migration occurs with just a few seconds of read-only downtime.

**Related Vitess documentation:**

- Sharding
- Horizontal sharding (Codelab)
- Sharding in Kubernetes (Codelab)

## Related tasks

In addition to the four steps discussed above, you might also want to do some or all of the following as your application matures.

### Data processing input / output

Hadoop is a framework that enables distributed processing of large data sets across clusters of computers using simple programming models.

Vitess provides a Hadoop InputSource that can be used for any Hadoop MapReduce job or even connected to Spark. The Vitess InputSource takes a simple SQL query, splits that query into small chunks, and parallelizes data reading as much as possible across database instances, shards, etc.

### Query log analysis

Database query logs can help you to monitor and improve your application's performance.

To that end, each vttablet instance provides runtime stats, which can be accessed through the tablet’s web page, for the queries the tablet is running. These stats make it easy to detect slow queries, which are usually hampered by a missing or mismatched table index. Reviewing these queries regularly helps maintain the overall health of your large database installation.

Each vttablet instance can also provide a stream of all the queries it is running. If the Vitess cluster is colocated with a log cluster, you can dump this data in real time and then run more advanced query analysis.

Contact: vitess@googlegroups.com  **·**  
Announcements  **·**  
© 2017 Vitess powered by Google Inc
